# Urinary NGAL as an Early Marker of Renal Dysfunction in Dogs with Heartworm Disease and Pulmonary Hypertension

**DOI:** 10.3390/ani15142003

**Published:** 2025-07-08

**Authors:** Noelia Costa-Rodríguez, Daniel Julio Vera-Rodríguez, Soraya Falcón-Cordón, Beatriz Regina Morales, Rodrigo Morchón, José Alberto Montoya-Alonso, Elena Carretón

**Affiliations:** 1 Internal Medicine, Faculty of Veterinary Medicine, Research Institute of Biomedical and Health Sciences (IUIBS), University of Las Palmas de Gran Canaria, 35413 Las Palmas de Gran Canaria, Spain; noelia.costa@ulpgc.es (N.C.-R.); daniel.vera@ulpgc.es (D.J.V.-R.); soraya.falcon@ulpgc.es (S.F.-C.); beatriz.morales@ulpgc.es (B.R.M.); elena.carreton@ulpgc.es (E.C.); 2 Zoonotic Diseases and One Health Group, Laboratory of Parasitology, Faculty of Pharmacy, University of Salamanca, 37007 Salamanca, Spain; rmorgar@usal.es

**Keywords:** *Dirofilaria immitis*, heartworm disease, canine, urinary NGAL, echocardiography, pulmonary hypertension, renal injury

## Abstract

Heartworm disease, caused by *Dirofilaria immitis*, often leads to pulmonary hypertension (PH), a serious cardiovascular complication in infected dogs. PH may impair renal function through hemodynamic and inflammatory mechanisms, even when traditional biomarkers such as serum creatinine and blood urea nitrogen (BUN) remain within normal ranges. This study aimed to assess urinary neutrophil gelatinase-associated lipocalin (uNGAL) levels in dogs naturally infected with *D. immitis*, with and without PH, to evaluate its potential as an early biomarker of renal dysfunction. Forty-two infected dogs were included and divided into two groups based on the presence (n = 14) or absence (n = 28) of PH, diagnosed via echocardiography. uNGAL concentrations were significantly higher in dogs with PH (mean 66.49 ± 6.67 ng/mL) compared to those without PH (mean 49.01 ± 14.48 ng/mL; *p* < 0.0001), despite normal creatinine and BUN values. No significant associations were found between uNGAL and sex, age, breed, or clinical signs. These findings suggest that uNGAL may serve as a sensitive biomarker of early renal impairment in dogs with heartworm disease and PH, even in the absence of overt azotemia, supporting its use in clinical evaluation and the monitoring of disease progression.

## Simple Summary

Heartworm disease is a serious condition in dogs caused by the parasite *Dirofilaria immitis*. In advanced stages, it can lead to pulmonary hypertension (PH), a condition that affects both the lungs and heart. PH may also harm the kidneys, even before standard tests like creatinine or urea indicate any abnormalities. In this study, we examined a protein called urinary NGAL (uNGAL), which is released by kidney cells in response to injury, to determine whether it could help detect early kidney damage in dogs with heartworms. We found that dogs with PH had higher levels of uNGAL than dogs without PH, despite having normal results on standard kidney tests. These findings suggest that uNGAL may be a valuable tool for detecting early renal damage and improving disease monitoring in affected dogs.

## 1. Introduction

Heartworm disease is a serious parasitic condition caused by the nematode *Dirofilaria immitis*. It is prevalent worldwide, particularly in regions with warm climates, and poses significant health risks to both domestic and wild canids [1,2]. The disease affects millions of dogs annually and, if left untreated, can lead to severe cardiovascular and pulmonary complications. Adult parasites primarily reside in the pulmonary arteries, where they induce pathological changes that reduce arterial elasticity and increase vascular resistance, ultimately resulting in precapillary pulmonary hypertension (PH) [3,4]. Precapillary PH is defined as elevated pressure within the pulmonary arterial vasculature and can arise as a complication of various underlying diseases [5]. Regardless of its etiology, sustained increases in pulmonary vascular pressure contribute to the progression of PH and eventually lead to varying degrees of right ventricular dysfunction and congestive heart failure (CHF) [6].

In addition to its cardiovascular effects, *D. immitis* infection is also associated with renal lesions, primarily caused by the deposition of immune complexes in the glomerular basement membrane. This leads to thickening and subsequent obstruction of the glomerular capillaries. Renal damage due to *Wolbachia* endosymbiont deposition and the presence of microfilariae have also been reported [7,8,9,10]. These processes damage the glomerular endothelium and promote the development of proliferative glomerulonephritis, which can manifest as proteinuria and, in some cases, progress to azotemia and hypoalbuminemia [11,12]. In human medicine, renal function is a strong, independent predictor of prognosis in patients with CHF [7]. In veterinary medicine, a similar relationship has been demonstrated in several studies [13,14,15]. However, in companion animals, the interplay between PH and renal injury remains complex and not yet fully understood [16]. Given that PH can progress to right-sided CHF if left untreated, and considering its high incidence in dogs with heartworm disease, further investigation into the potential relationship between these conditions is warranted.

Doppler echocardiography is the diagnostic method of choice for detecting PH in veterinary medicine, offering a non-invasive and accessible means of estimating pulmonary arterial pressure [5]. Regarding renal function, traditional markers such as serum creatinine and blood urea nitrogen (BUN) concentrations are still the most commonly used, despite their limitations. These markers are late indicators of kidney disfunction and cannot distinguish between functional and structural damage [17,18]. While creatinine and BUN levels typically rise only after approximately 60–70% of renal function has been lost, symmetric dimethylarginine (SDMA)—an endogenous biomarker of renal excretory function—can increase earlier, after about a 40% reduction in glomerular filtration rate, making it a more sensitive indicator for early detection [17,19].

Recent advances have identified neutrophil gelatinase-associated lipocalin (NGAL) as a promising biomarker of kidney injury in veterinary medicine. NGAL, a member of the lipocalin family, is expressed by neutrophils and various other cells, including renal tubular epithelial cells, pulmonary cells, and cardiomyocytes. It is rapidly released into the bloodstream and urine in response to tubular injury [20]. Although its role has been widely studied in other clinical contexts [20,21,22,23,24], the potential of urinary NGAL (uNGAL) as a biomarker in canine heartworm disease—and its association with PH—remains unexplored.

This study aimed to evaluate uNGAL concentrations as an early marker of renal dysfunction in dogs with heartworms, and to determine whether the presence of PH influences this parameter. If confirmed, uNGAL could serve as a valuable tool for assessing renal function and improving the clinical management of affected dogs.

## 2. Materials and Methods

This prospective study included 42 dogs naturally infected with *D. immitis* that presented to the Veterinary Teaching Hospital of the University of Las Palmas de Gran Canaria (ULPGC) between September 2022 and September 2023. A detailed record was maintained for each animal, including identification, age, sex, breed, and the presence or absence of clinical signs at diagnosis. All dogs resided in heartworm-endemic areas, ensuring homogeneity in exposure risk. Heartworm infection was diagnosed using a commercial immunochromatographic test kit (Uranotest Dirofilaria, Urano Vet SL, Barcelona, Spain). In addition, routine hematological and biochemical analyses were performed, including renal parameters (BUN and creatinine).

Inclusion criteria required dogs to test positive for *D. immitis* antigen without prior prophylactic or adulticidal treatment. Exclusion criteria ruled out animals with pre-existing cardiorespiratory conditions that could independently cause PH—such as chronic degenerative valve disease, dilated cardiomyopathy, congenital heart defects, or chronic respiratory diseases—as well as unrelated renal disorders.

All dogs underwent echocardiographic examination, including Doppler ultrasound, using multifrequency probes (2.5–10 MHz) on a Vivid Iq^®^ system (General Electric, Boston, MA, USA). Examinations were performed by a member of the research team with six years of exclusive experience in small animal cardiorespiratory medicine, and were always conducted by the same investigator to ensure consistency. Dogs were examined while conscious and gently restrained in right and left lateral recumbency under continuous electrocardiographic monitoring. The presence or absence of PH was assessed following the guidelines of the American College of Veterinary Internal Medicine (ACVIM) [5]. Standard parameters such as maximum tricuspid regurgitation velocity (TRV) and systolic pulmonary artery flow were recorded. Additionally, the right pulmonary artery distensibility index (RPAD index) was measured as previously described [25], and pulmonary artery diameters were calculated accordingly [25,26,27]. TRV was measured using apical four-chamber and left cranial transverse views. Special care was taken to align the cursor parallel to the direction of flow, optimize gain settings, and measure the regurgitant jet at the dense outer edge of the velocity profile, avoiding fine linear signals (the “feathered edge”) [5]. For each parameter, three consecutive cardiac cycles were recorded. Parasite burden was evaluated via echocardiography using a previously established scoring system [28], which classified relative worm burden on a scale from 1 to 4. Scores of 1–2 were considered low (indicating no visible parasites or only a few echoes in the distal segment of the right pulmonary artery), while scores of 3–4 were considered high (indicating worm echoes occupying the right pulmonary artery or extending into the main pulmonary artery).

Urine samples were collected via ultrasound-guided cystocentesis. Semi-quantitative analysis was performed using two different commercial test strips (Uranotest 11C and Uranotest 2AC; Urano Vet SL, Barcelona, Spain), which were analyzed with the automatic semi-quantitative analyzer Uranotest Reader^®^ (Urano Vet SL, Barcelona, Spain), according to the manufacturer’s instructions. The remaining urine was stored at −80 °C until further analysis. Urinary NGAL (uNGAL) concentrations were measured using a commercial sandwich ELISA kit (Dog NGAL ELISA Kit, BIOPORTO Diagnostics, Hellerup, Denmark), with reference values for healthy dogs reported as <60 ng/mL, according to the manufacturer’s specifications. The assay was calibrated and validated in the laboratory of the Department of Animal Pathology, Faculty of Veterinary Medicine, ULPGC. For validation purposes, stored serum samples from 20 healthy dogs were also analyzed. Results were expressed as urinary NGAL concentrations (uNGAL) in ng/mL.

Data were analyzed using SPSS Base 29.0 software for Windows (SPSS Inc./IBM, Chicago, IL, USA). Categorical variables were summarized using frequencies and percentages, while continuous variables were described using mean, standard deviation, median, and interquartile range. Differences in continuous variables between groups were evaluated using either the Mann–Whitney or Kruskal–Wallis tests (non-parametric), or Student’s t-test or ANOVA (parametric), depending on the normality of the data as assessed by the Shapiro–Wilk test. Differences in categorical variables were analyzed using Pearson’s Chi-squared test. All statistical comparisons were accompanied by effect size estimates to aid interpretation: Cramer’s V for categorical variables and Cohen’s d for continuous variables. When statistically significant differences were found using the Kruskal–Wallis test, post hoc pairwise comparisons were conducted using the Mann–Whitney U test with Bonferroni correction. Receiver operating characteristic (ROC) curve analyses were performed to determine the optimal cutoff values, using a reference threshold of 60 ng/mL. Sensitivity and specificity were assessed based on the cutoff points that maximized diagnostic accuracy. A *p*-value < 0.05 was considered statistically significant.

Ethical approval was not required for this study, as all blood samples were collected as part of routine diagnostic and monitoring procedures and were subsequently made available for research. All owners were informed about the study and provided written informed consent prior to participation. The study was conducted in accordance with current Spanish and European legislation on animal protection.

## 3. Results

Based on echocardiographic evaluation, dogs were divided into two groups: Group A (n = 28), consisting of dogs without PH, and Group B (n = 14), consisting of dogs with PH (Table 1).

Most of the studied dogs were purebred (52.4%, n = 22), while 47.6% (n = 20) were mongrels. The most common pure breeds included the Canarian Hound, German Shepherd, Cocker Spaniel, and American Pit Bull Terrier. Males represented 54.8% (n = 23) of the population, while females accounted for 45.2% (n = 19). The age range of the studied dogs was 1 to 10 years (mean 4.6 ± 2.7 years). No statistically significant differences were observed in breed or sex in relation to the presence or absence of PH. Similarly, age comparison between dogs with and without PH using the Mann–Whitney U test revealed no statistically significant difference between groups (U = 0.618; *p* = 0.196).

uNGAL concentrations were significantly higher in Group B (66.49 ± 6.67 ng/mL) compared to Group A (49.01 ± 14.48 ng/mL), with a statistically significant difference (*p* < 0.0001). Based on reference values, 78.5% (n = 11) of Group B dogs had uNGAL concentrations above the reference threshold, compared to only 7% (n = 1) in Group A. No statistically significant differences in uNGAL concentrations were found with respect to age, sex, or breed.

**Figure 1:**
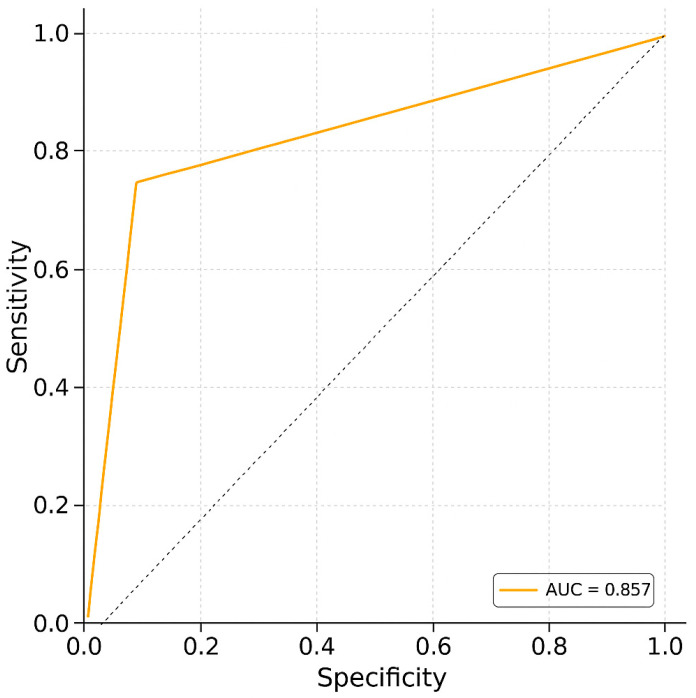
Receiver operating characteristic (ROC) curve analysis. The ROC curve illustrates the trade-off between sensitivity and specificity for the diagnostic test. The area under the curve (AUC) is 0.857, reflecting a strong ability to discriminate between positive and negative cases (sensitivity: 78.6%; specificity: 75%).

Receiver operating characteristic (ROC) curve analysis was conducted to evaluate the ability of uNGAL to discriminate between dogs with and without PH (Figure 1). The analysis yielded an area under the curve (AUC) of 0.857, indicating good diagnostic accuracy. The optimal cutoff value was identified as 60 ng/mL and was statistically significant (*p* = 1.07 × 10^−7^). Cohen’s d was 46.20, indicating a very large effect size and supporting the strong discriminative power of uNGAL in this context.

Microfilaremia was detected in 66.7% (n = 28) of dogs, while 33.3% (n = 14) tested negative for circulating microfilariae. When stratified by PH status, 57.14% (n = 16) of dogs in Group A and 85.71% (n = 12) in Group B were microfilaremic. Although the proportion was higher in Group B, the difference was not statistically significant (U = 0.593; *p* = 0.336). Additionally, no significant differences in uNGAL concentrations were observed between microfilaremic and amicrofilaremic dogs.

**Figure 2:**
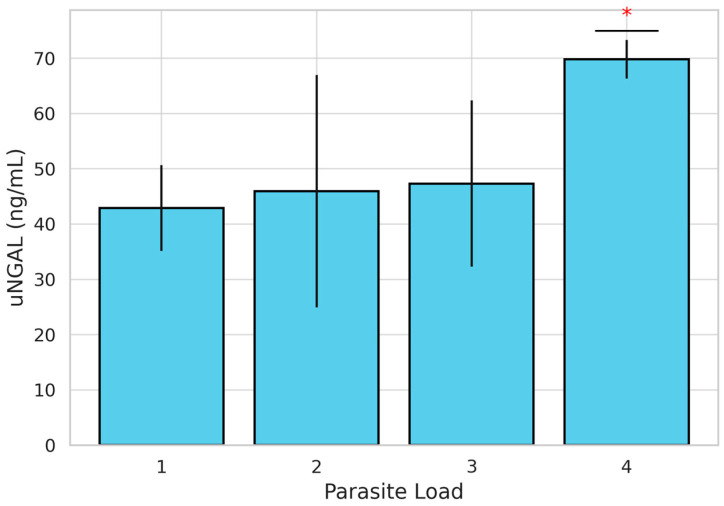
Bar graph showing mean uNGAL concentrations (ng/mL) ± standard deviation in dogs naturally infected with *Dirofilaria immitis*, stratified by parasite burden score (1 to 4). A statistically significant increase in uNGAL was observed in dogs with a score of 4 compared to those with a score of 3 (*) (*p* = 0.007, Mann–Whitney U test with Bonferroni correction).

Parasitic burden was also evaluated, with 22 dogs classified as having a low burden (scores 1 and 2) and 20 dogs classified as having a high burden (scores 3 and 4). When stratified by PH status, 39.3% (n = 11) of dogs in Group A and 64.3% (n = 9) in Group B showed a high parasite burden. However, the difference between groups was not statistically significant (χ2 =1.444; *p* = 0.230). When comparing uNGAL concentrations between high- and low-parasite-burden groups, although concentrations were higher in Group B, no statistically significant differences were found (Table 2). Nevertheless, the evaluation of uNGAL concentrations across individual parasite burden scores using the Kruskal–Wallis test revealed statistically significant differences (H = 12.087; *p* = 0.007). To further explore these differences, a post hoc pairwise comparisons using the Mann–Whitney U test with Bonferroni correction was performed. These analyses showed that uNGAL concentrations were significantly higher in dogs with a parasite burden score of 4 compared to those with a score of 3 (*p* = 0.007). No other pairwise comparisons between scores (1 vs. 2, 1 vs. 3, etc.) reached statistical significance (Figure 2). Dogs with a parasite burden score of 4 showed the highest mean uNGAL concentration (n = 6; 69.83 ng/mL), followed by scores of 3 (n = 14; 47.85 ng/mL), 2 (n = 19; 46.36 ng/mL), and 1 (n = 3; 41.08 ng/mL), suggesting a positive association between parasitic burden and uNGAL levels.

Clinical signs were observed in 19 dogs at the time of examination or as reported in their clinical history. The clinical signs included cough, dyspnea, weight loss, exercise intolerance, pale mucous membranes, syncope, and ascites. Only a single dog had R-CHF, which precluded additional subgroup analysis. Although 64.3% (n = 9) of dogs of Group B showed clinical signs vs. 35.7% (n = 10) in Group A, no statistically significant associations were found between the presence of clinical signs and PH, or between clinical signs and uNGAL concentrations (Table 2). None of the dogs presented with vena cava syndrome.

No dogs showed abnormal serum creatinine or BUN levels. Overall, 7.1% (n = 3) of dogs showed proteinuria (UPC > 0.5) and 26.2% (n = 11) had borderline proteinuria (UPC 0.2–0.5). When stratified by PH status, 21.43% (n = 6) of Group A dogs and 57.14% (n = 8) of Group B dogs showed proteinuria or borderline proteinuria. The relationship between proteinuria and PH was assessed using the Chi-squared test. Although uNGAL concentrations tended to be higher in proteinuric dogs, no statistically significant differences were observed (χ^2^ = 0.98; *p* = 0.321) (Table 2). A moderate positive correlation was observed between proteinuria and uNGAL concentrations (Spearman’s ρ = 0.489); however, this correlation did not reach statistical significance (*p* = 0.076).

## 4. Discussion

Pulmonary hypertension is a common and serious complication of canine heartworm disease and one of the main contributors to clinical signs in infected dogs. If left untreated, it can progress to right-sided heart failure, severely compromising the animal’s health [29]. The development of pulmonary microvascular dysfunction increases vascular resistance and right ventricular afterload, ultimately leading to progressive cardiac deterioration [29,30]. Although PH is more likely in chronic infections, typically in older dogs, no significant age differences were observed between dogs with and without PH in this study, consistent with previous reports [31,32,33].

As cardiac dysfunction progresses, central venous pressure rises, reducing effective renal perfusion and increasing interstitial and hydrostatic pressures in Bowman’s capsule. These changes impair both glomerular and tubular function [34,35], potentially leading to subclinical kidney injury. In such cases, tubular epithelial cells may release NGAL in response to damage and inflammation [22,23].

In human medicine, elevated NGAL levels have been associated with PH. For instance, in patients with acute coronary syndrome, higher uNGAL levels were found in those with PH, suggesting uNGAL as a useful biomarker for detecting cardiac complications, even in the absence of overt renal dysfunction [36]. In pediatric patients with congenital heart disease, NGAL has also been proposed as a marker of inflammation and vascular remodeling in the context of PH [37]. Experimental studies have shown that NGAL contributes to PH progression by inhibiting apoptosis in pulmonary artery smooth muscle cells, thereby promoting proliferation and vascular thickening [38]

The findings of this study may reflect early tubular injury resulting from venous congestion, as well as chronic systemic inflammation secondary to heartworm disease and PH. This pattern resembles that observed in other canine diseases such as leishmaniasis [22,32]. Although only one dog in our cohort showed signs of right-sided CHF, it is well documented that renal venous congestion and impaired perfusion can occur even without overt clinical signs (e.g., ascites), due to elevated right atrial and central venous pressures [39]. This can lead to subclinical renal injury before clinical CHF is apparent. Additionally, inflammation induced by heartworms likely contributes to pulmonary vascular remodeling and renal injury via immune complex deposition and systemic cytokine activation. Prior studies have identified acute-phase proteins in heartworm-infected dogs, particularly those with PH [40,41]. Moreover, *D. immitis* induces renal changes through immune complex deposition, microfilaremia, and the presence of *Wolbachia* bacteria [7,9]. Glomerular lesions associated with heartworm disease include glomerulosclerosis, chronic interstitial nephritis, and renal amyloidosis [10,42,43,44], and endothelial injury from immune complexes may result in proliferative glomerulonephritis [11,45]. Although this study cannot fully differentiate among these mechanisms, they likely act synergistically and should be jointly considered in the pathogenesis of renal injury in heartworm disease with PH.

Significantly, uNGAL levels were elevated in dogs with PH, even in the absence of abnormal serum creatinine or BUN, reinforcing its role as an early and sensitive biomarker of tubular injury. This suggests that uNGAL can detect renal involvement earlier than conventional functional markers. Additionally, a trend toward a positive association between uNGAL and proteinuria, although not statistically significant, may indicate combined tubular and glomerular damage. These findings support the potential clinical relevance of uNGAL for detecting early renal impairment in dogs with advanced *D. immitis* infection and PH.

A higher proportion of dogs with PH exhibited proteinuria or borderline proteinuria (57.1%) compared to those without PH (21.4%), although this difference was not statistically significant. Additionally, a moderate positive correlation was observed between uNGAL and proteinuria; while not reaching statistical significance, this trend may be relevant for future studies, especially considering the limited sample size. Moreover, it remains biologically plausible. Proteinuria primarily reflects glomerular permeability defects, while uNGAL indicates tubular cell damage. Sustained proteinuria can exert a toxic effect on proximal tubular cells through mechanisms like endocytic overload, oxidative stress, and cytokine induction, all of which promote NGAL expression and release [46]. Conversely, the hemodynamic and inflammatory environment associated with PH may simultaneously injure glomeruli and tubules [47,48]. In patients with chronic kidney disease, persistent proteinuria has been shown to induce tubulointerstitial inflammation and fibrosis [49,50]. Given the known interrelation between glomerular and tubular injury as well as the moderate correlation observed in this study, the authors postulate that the lack of statistical significance found in this study may be secondary to a small sample size and further research with larger study populations is needed. Previous research has demonstrated that dogs with PH due to heartworm disease show greater proteinuria [11], reinforcing the idea that both hemodynamic and inflammatory mechanisms contribute to renal damage. Proteinuria often precedes overt signs of renal dysfunction, underscoring its value as an early marker [51]. Thus, the coexistence of proteinuria and elevated uNGAL likely reflects dual glomerular and tubular injury, driven by the inflammatory and hemodynamic stress of PH.

No significant differences in uNGAL levels were found between microfilaremic and amicrofilaremic dogs. While this study did not reveal an association, the limited sample size may have hindered the detection of true differences, and larger studies are needed to further assess the impact of microfilaremia on renal biomarkers like uNGAL.

Although SDMA is widely recognized as a sensitive marker of early glomerular filtration rate decline, it was not included in this study because its diagnostic performance had already been assessed in a previous study [11]. That study found no significant differences in SDMA between healthy dogs and those naturally infected with *D. immitis*, including those with PH. These results suggest that SDMA alone may have limited utility for identifying early renal dysfunction in this context. Nevertheless, future research incorporating both glomerular and tubular biomarkers—such as SDMA and uNGAL—in larger and longitudinally monitored populations may help clarify their combined diagnostic and prognostic utility. Therefore, the combined use of both markers may provide complementary insights into different stages and compartments of renal injury.

Parasite burden also influenced uNGAL concentrations. Although no significant differences were found using a simple high/low classification, statistically significant differences emerged when parasite burden was assessed using a 4-point scoring system [28]. Dogs with a score of 4 had the highest uNGAL levels, supporting the hypothesis that higher worm loads provoke more severe renal damage through greater antigenic stimulation, immune complex formation, and inflammation [52,53,54,55]. The parasites and their associated components (including *Wolbachia*) not only contribute to PH via vascular remodeling but also directly promote renal injury via persistent antigenic stimulation, immune complex formation and deposition, and the activation of systemic pro-inflammatory pathways [1,3,29]. These mechanisms may lead to both glomerular and tubular injury, independent of hemodynamic compromise, and should be considered as key contributors to renal pathology in heartworm-infected dogs, particularly in those with high worm burdens.

Interestingly, symptomatic dogs did not show statistically significant elevations in uNGAL levels. This discrepancy with previous studies, in which dogs with heartworm and PH were significantly more symptomatic [26,31,32], may again be explained by the small sample size and may also have influenced the lack of significance with the uNGAL results.

This study has several limitations. The relatively small number of dogs with PH (n = 14) may have reduced statistical power, especially for subgroup analyses (e.g., proteinuria, clinical signs, microfilaremia, and the small number of dogs with overt right-sided CHF). Though non-significant, observed trends—such as higher rates of proteinuria and symptoms in PH dogs—may still be clinically relevant. Future studies with larger, more balanced samples are necessary to confirm these findings and better assess the relationships between PH, renal damage, and systemic manifestations. Although the 60 ng/mL cutoff for uNGAL was based on manufacturer-provided values from healthy dogs, ROC curve analysis in this study supported its diagnostic value for identifying PH among heartworm-infected dogs. However, this threshold has not been validated across broader canine populations, and additional studies are needed to define disease-specific reference intervals. Lastly, infection chronicity was not directly measured, which may have influenced the results, as PH and renal complications evolve over time [4,11,56]. Furthermore, proteinuria was assessed using a semi-quantitative automated dipstick analysis rather than UPC ratio. While standardized and machine-read to reduce variability, dipstick testing is less accurate and may misestimate proteinuria.

## 5. Conclusions

Dogs with heartworm disease and PH exhibited increased uNGAL concentrations, suggesting the presence of subclinical renal involvement even in the absence of elevations in conventional markers such as creatinine and BUN. The observed trends highlight the potential utility of uNGAL as an early biomarker of renal dysfunction in dogs with cardiovascular complications of *Dirofilaria immitis* infection. Therefore, uNGAL may serve as a valuable tool for the early detection of renal impairment in dogs infected with *D. immitis*, particularly in those with advanced cardiovascular involvement, thereby facilitating the implementation of preventive therapeutic strategies.

Given that renal involvement in heartworm disease remains a poorly explored area with no standardized clinical protocols for its diagnosis, staging, or treatment, further investigation is essential. Particular attention should be paid to renal function in dogs with PH, and the continued evaluation of the diagnostic and prognostic value of biomarkers such as uNGAL is strongly recommended.

## Figures and Tables

**Table 1:** Echocardiographic parameters measured in dogs with and without pulmonary hypertension. Values are expressed as mean (minimum–maximum). Abbreviations: FS: Fractional shortening; EF: Ejection fraction; LVIDd: Left ventricular internal diameter in diastole; RVDd: Right ventricular end-diastolic diameter; PA Vmax: Peak velocity of the pulmonary artery; PT:Ao: Pulmonary trunk-to-aorta ratio; TAPSE: Tricuspid annular plane systolic excursion; RAV: Right atrial volume; RPAD index: Right pulmonary artery distensibility index; AT: Right ventricular acceleration time; DT: Deceleration time; ET: Right ventricular ejection time; TRV: Maximum tricuspid regurgitation velocity.

Dogs with Pulmonary Hypertension (n = 14)	
Parameter	Values
FS (%)	36.34 (29.31–42.94)
EF (%)	65.27 (54.70–72.47)
LVIDd (cm)	0.55 (0.52–0.60)
RVDd (cm)	0.92 (0.77–1.17)
IVSd (cm)	0.92 (0.80–1.12)
PA Vmax (m/s)	0.80 (0.68–0.88)
PT:Ao	1.04 (1.00–1.12)
TAPSE (cm)	1.46 (1.05–1.54)
RAV (ml)	4.93 (1.61–6.41)
RPAD index	25.67 (7.93–33.01)
AT (ms)	60.30 (60.30–80.40)
DT (ms)	126.65 (118.65–144.05)
ET (ms)	160.80 (32.62–185.93)
AT/ET	0.16 (0.11–0.18)
TRV m/s	3.33 (3.00–3.56)
**Dogs Without Pulmonary Hypertension (n = 28)**	
**Parameter**	**Values**
FS (%)	37.70 (31.81–39.77)
EF (%)	65.90 (58.80–72.09)
LVIDd (cm)	0.57 (0.47–0.69)
RVDd (cm)	0.96 (0.83–1.09)
IVSd (cm)	1.05 (0.94–1.15)
PA Vmax (m/s)	0.82 (0.73–0.92)
PT:Ao	0.99 (0.96–1.05)
TAPSE (cm)	1.35 (1.13–1.50)
RAV (ml)	5.55 (3.44–10.37)
RPAD index	30.27 (21.65–44.57)
AT (ms)	64.00 (60.30–85.42)
DT (ms)	117.25 (100.50–132.32)
ET (ms)	33.95 (24.95–152.43)
AT/ET	0.14 (0.12–0.18)
TRV m/s	1.10 (0.97–1.24)

**Table 2:** uNGAL concentrations based on different classifications of the study dogs. Data are presented as mean ± standard deviation. Statistically significant results (*p* < 0.05) are marked with an asterisk. PH = pulmonary hypertension; Microfilaremia = presence of microfilariae in peripheral blood; Proteinuria/Borderline Proteinuria = presence of proteins in urine. Groups were categorized according to high or low parasite burden as described [28].

	Concentrations of uNGAL		*p*-Value
	Present	Absent	
PH	66.49 ± 6.67 ng/mL (n = 14)	49.01 ± 14.48 ng/mL (n = 28)	<0.0001 *
Microfilaremia	51.41 ± 18.89 ng/mL (n = 28)	46.66 ± 15.92 ng/mL (n = 14)	0.588
Symptoms	62.09 ± 9.25 ng/mL (n = 19)	43.70 ± 18.12 ng/mL (n = 23)	0.427
Proteinuria/Borderline Proteinuria	62.09 ± 9.25 ng/mL (n = 14)	43.70 ± 18.12 ng/mL (n = 28)	0.076
	High	Low	
Parasite Burden	54.83 ± 16.41 ng/mL (n = 20)	45.64 ± 19.97 ng/mL (n = 22)	0.154

## Data Availability

All data generated or analyzed during this study are included in this article. The datasets used and/or analyzed during the present study are available from the corresponding author upon reasonable request.
